# Prevalence and Antibiotic Resistance Patterns of *Helicobacter pylori* Infection in Koh Kong, Cambodia

**DOI:** 10.31557/APJCP.2020.21.5.1409

**Published:** 2020-05

**Authors:** Natsuda Aumpan, Ratha-Korn Vilaichone, Pornpen Gumnarai, Likhasit Sanglutong, Thawee Ratanachu-ek, Varocha Mahachai, Yoshio Yamaoka

**Affiliations:** 1 *Gastroenterology Unit, Department of Medicine, Faculty of Medicine, Thammasat University Hospital, Pathumthani, Thailand. *; 2 *Digestive diseases Research Center (DRC), Thammasat University, Pathumthani, Thailand. *; 3 *Department of Medicine, Chulabhorn International College of Medicine (CICM) at Thammasat University, Pathumthani, Thailand. *; 4 *Department of Biochemistry, Faculty of Medicine, Thammasat University, Pathumthani, Thailand. *; 5 *Department of Surgery, Rajavithi Hospital, Bangkok, Thailand. *; 6 *Gastrointestinal and Liver Center, Bangkok Medical Center, Bangkok, Thailand. *; 7 *Department of Environmental and Preventive Medicine, Oita University Faculty of Medicine, Yufu, Japan. *

**Keywords:** Antibiotic resistance, Helicobacter pylori, Cambodia

## Abstract

**Background:**

Gastric cancer, which is the leading cause of cancer mortality in Cambodia, can be prevented by *Helicobacter pylori *(*H. pylori*) eradication. There is limited data about *H. pylori* strains in Cambodia. This study aimed to evaluate *H. pylori* prevalence and antibiotic resistance in Koh Kong, Cambodia.

**Methods:**

118 Cambodian dyspeptic patients were scheduled to enter this study and 58 were enrolled between July and September 2019. All patients underwent upper GI endoscopy. 3 gastric biopsies were obtained for rapid urease test, *H. pylori* culture with E-test and GenoType® HelicoDr (Hain Lifescience factory, Germany). 3-mL blood sample was collected for CYP2C19 genotyping.

**Results:**

58 subjects were enrolled (40 females, 18 males, mean age 43.8 years). Overall *H. pylori* prevalence was 31.0%. Antibiotic resistance rates were 78.6% for metronidazole, 50.0% for fluoroquinolones, and 27.8% for clarithromycin. There was no amoxicillin and tetracycline resistance. More than half of *H. pylori* strains (57.1%) were multidrug-resistant. Most (35.7%) were resistant to metronidazole and quinolone. Poor, intermediate and rapid metabolizers were 5.5%, 38.9% and 55.6%, respectively.

**Conclusions:**

*H. pylori* infection remains common infection in Cambodia. High prevalence of clarithromycin, metronidazole, levofloxacin and multidrug-resistant *H. pylori* is still major problems in Cambodia. Treatment regimens without clarithromycin and quinolone such as 14-day bismuth-based quadruple therapy might be an appropriate choice for *H. pylori* eradication in this particular area.

## Introduction

*Helicobacter pylori* (*H. pylori*) is a gram-negative, spiral-shaped bacterium causing persistent gastric mucosal infection. Various *H. pylori*-associated conditions range from gastritis and gastroduodenal ulcers to more severe diseases including mucosa–associated lymphoid tissue (MALT) lymphoma, and gastric cancer (McColl, 2010; Poonyam et al., 2019). Considered as carcinogens, *H. pylori* promote non-cardia gastric carcinogenesis through chronic gastric mucosal inflammation in Correa’s precancerous cascade (Correa and Piazuelo, 2012; Rugge et al., 2017). *H. pylori* eradication resulted in reduced gastric cancer incidence, regression of gastric premalignant lesion, and healing of peptic ulcers (Takenaka et al., 2007; Vannella et al., 2011; Hosking et al., 1994). However, there are several factors affecting eradication success rate such as host genetics, bacterial virulence, and environmental factors (Kuster et al., 2006; Yamaoka and Graham, 2014). Recently, antibiotic-resistant strains have been increasing worldwide and subsequently had an effect on treatment failure rate (Savoldi et al., 2018). Southeast Asian countries are currently facing major problems about drug-resistant *H. pylori* and appropriate treatment regimen for each country should be reviewed depending on antimicrobial susceptibility testing (Vilaichone et al., 2018).

Cambodia is the Southeast Asian sovereign state bordered by Thailand to its northwest, Laos to its northeast, and Vietnam to its east. Koh Kong province is located in the southwestern part of Cambodia. Its geography is characterised by mountains, rainforests and coastal areas. Koh Kong’s population has been increasing since the improvement of infrastructure in 2002. According to global cancer statistics 2018, gastric cancer is the eighth leading cause of cancer-related deaths in Cambodia (International Agency for Research on Cancer, 2018). As diagnostic tools and treatment facilities are not easily accessible for Cambodian people, health care providers need to implement cancer prevention strategies to decrease the incidence of specific cancers (Eav et al., 2012). Regarding as one of preventable cancers, most of gastric cancers associated with *H. pylori* infection can be prevented by bacterial eradication (Mahachai et al., 2016). However, there has been limited research determining the prevalence of *H. pylori* infection and antibiotic susceptibility testing cannot be performed in Cambodia (Quach et al., 2018; Vilaichone et al., 2018). 

Nowadays, the information about *H. pylori* infection in Cambodia is still lacking. This study aimed to evaluate prevalence and antibiotic resistance patterns of *H. pylori* infection in Koh Kong, Cambodia. The result of this study could be used for developing appropriate regimen for *H. pylori* treatment in Cambodia. 

## Materials and Methods

*Patients*

118 Cambodian patients with dyspepsia were scheduled to enter this study and 58 patients were enrolled between July and September 2019. All 58 patients (40 females, 18 males, mean age 43.8 years) underwent upper GI endoscopy and 3 gastric biopsies were obtained for rapid urease test (Pronto Dry®, Eisai, Thailand), *H. pylori* culture with Epsilometer test (E-test) and GenoType® HelicoDr (Hain Lifescience factory, Nehren, Germany). 3 mL of blood sample was collected for CYP2C19 genotyping. The exclusion criteria were as follows; patients receiving H2 receptor antagonists, proton pump inhibitor (PPI), bismuth compound, and antimicrobial agents within 4 weeks prior to the study, using NSAIDs and anticoagulant, having history of stomach surgery, or having significant comorbidities such as renal failure, advanced cirrhosis, advanced or metastatic cancer, or cardiac arrhythmia, immunocompromised patients, pregnant women, and breastfeeding women. Informed consent was obtained from study volunteer before upper GI endoscopy was performed.

*Rapid urease test*

This is a diagnostic test for detection of urease-secreting bacteria. After the gastric biopsy had been placed in the center of the well containing urea and pH indicator, the test was kept at room temperature for 60 minutes. Urease produced by *H. pylori* in gastric specimen could hydrolyze urea to ammonia and raise the pH of the medium. Consequently, the color of pH indicator changed from yellow to pink or red as a positive result. 

*H. pylori culture*

Antral biopsies obtained for *H. pylori* culture were contained in transport media in the Eppendorf tubes at -40°C in the freezer during 446-kilometer transportation from Cambodia to Thammasat University Hospital, Pathumthani, Thailand. In the laboratory, gastric biopsies were minced in the broth. We used a sterile heated wire loop to streak the fluid mixture on a Mueller Hinton - Agar medium. The media were then placed in the candle jar and incubated at 37°C in microaerophilic condition. *H. pylori* became visible as translucent colonies on blood agar approximately 3 to 5 days after incubation. Gram staining of these bacteria demonstrated curved gram-negative rods with positive oxidase, catalase, and urease tests.

*Antimicrobial susceptibility testing*

The Epsilometer test (E-test) directly quantifies susceptibility of bacteria to specific antibiotics by determining the minimum inhibitory concentrations (MICs) of antibiotics including amoxicillin (AMX), clarithromycin (CLR), metronidazole (MNZ), tetracycline (TET), and levofloxacin (LVX). An E-test strip with antibiotic was placed on a previously inoculated plate. On the subsequent 3 to 5 days, the MIC value could be defined as the intersection of an ellipse and the scale on the E-test strip. The MIC is the lowest concentration of antibiotics that can inhibit visible bacterial growth. *H. pylori* was considered resistant when MIC values were > 0.12 μg/mL for AMX, > 0.5 μg/mL for CLR, > 8 μg/mL for MNZ, > 1 μg/mL for LVX, and > 1 μg/mL for TET. 

*GenoType® HelicoDr test*

GenoType® HelicoDr test is a molecular test for detection of genetic mutations resulting in CLR and quinolone resistance. In our study, the tests were used in patients with negative *H. pylori* culture to determine whether they had CLR or quinolone-resistant strains. First, *H. pylori* genomic DNAs were extracted from gastric biopsies using QIAamp DNA Mini Kit (QIAGEN, Inc. Santa Clarita, CA, USA). The extracted DNAs were then amplified by polymerase chain reaction (PCR). If amplified DNA regions involving clarithromycin (rrl gene) and fluoroquinolone (gyrA gene) resistance were present, specific probes would bind them during hybridization. The positive band was visibly detected by the colorimetric assays (Cambau et al., 2009; Vannarath et al., 2016).

*Statistical analysis*

All data were analysed by using SPSS version 22 (SPSS Inc., Chicago, IL, USA). The demographic data were analysed by using the Fisher’s exact test, and Chi-square test where appropriate. Statistical significance was defined as a two-tailed p-value cut point of less than 0.05. 

## Results

Total of 58 dyspeptic Cambodian patients were enrolled in the study. There were 18 males (31.0%) and 40 females (69.0%) with the mean age of 43.8 years. Median duration of dyspepsia was 36 months. Patients mostly had gastritis (91.4%) on endoscopic findings, whereas 8.6% had peptic ulcer diseases. The prevalence of *H. pylori* infection was 31.0%. Baseline characteristics were not different between groups of positive and negative *H. pylori* infection. Demographic data including gender, age, comorbidities, and endoscopic findings were demonstrated in Table 1.

Of 18 patients with *H. pylori* infection, 14 patients could achieve *H. pylori* cultures. Another 4 patients subsequently performed HelicoDr tests. Antibiotic resistance rates were 78.6% for metronidazole, 50.0% for fluoroquinolones, and 27.8% for clarithromycin as demonstrated in Table 2. There was no amoxicillin and tetracycline resistance in this study. More than half of *H. pylori* strains (57.1%) were multidrug-resistant and most of them (35.7%) were resistant to MNZ and quinolones. *H. pylori* strains with triple drug resistance to CLR, MNZ, and quinolones were found in 14.3% of all patients. CYP2C19 genotyping was performed and revealed 5.5%, 38.9% and 55.6% for poor, intermediate and rapid metabolizers, respectively as demonstrated in Table 3.

**Table 1:** Demographic Data, Comorbidities and Endoscopic Findings between Groups

Groups	Total	*H. pylori* positive	*H. pylori* negative	*P*-value
Number of patients	58 (100%)	18 (31.0%)	40 (69.0%)	-
Gender				0.386
Male	18 (31.0%)	7 (38.9%)	11 (27.5%)	
Female	40 (69.0%)	11 (61.1%)	29 (72.5%)	
Mean age ± SD (yr)	43.8 ± 12.2	46.8 ± 11.2	42.4 ± 12.5	0.202
Range	15 - 62	20 - 62	15 - 61	
Comorbidities				
None	47 (81.0%)	14 (77.8%)	33 (82.5%)	0.724
Hypertension	8 (13.8%)	2 (11.1%)	6 (15.0%)	1.000
Diabetes mellitus	2 (3.4%)	1 (5.6%)	1 (2.5%)	0.528
Median duration of symptoms (months)	36	42	36	0.680
IQR	12 - 75	15 - 111	12 - 69	
Endoscopic findings				0.641
Gastritis	53 (91.4%)	16 (88.9%)	37 (92.5%)	
Peptic ulcer diseases	5 (8.6%)	2 (11.1%)	3 (7.5%)	

**Table 2:** Prevalence of Antibiotic-Resistant *H. pylori *Strains

*H. pylori* strains	Number of patients (%)
No antibiotic resistance	7.1
Antibiotic resistance	
Amoxicillin (AMX)	0
Tetracycline (TET)	0
Clarithromycin (CLR)	27.8
Metronidazole (MNZ)	78.6
Ciprofloxacin (CIP)	50.0
Levofloxacin (LVX)	50.0
>1 antibiotic resistance	
CLR and MNZ	7.1
MNZ, CIP, and LVX	35.7
CLR, MNZ, CIP, and LVX	14.3

*(n = 14)

**Table 3:** CYP2C19 Genotype

CYP2C19 genotype	Number of patients (%)
Rapid metabolizer	10 (55.6)
Intermediate metabolizer	7 (38.9)
Poor metabolizer	1 (5.5)

*(n = 18)

## Discussion

Cambodia has been reckoned as a mysterious country with vulnerable economic growth despite encouraged international tourism since the late 1990s. The healthcare service and medical research are still under-subsidized requiring international collaboration to refine the country’s healthcare system. Although there was limited access to healthcare service and antibiotics were presumed to be used infrequently, our study demonstrated extremely high prevalence of multidrug-resistant *H. pylori* strains in Cambodia. The overall prevalence of *H. pylori* infection in our study was 31.0% which was quite low compared to neighbouring countries’. Since Thai-Koh Kong bridge was built and Chinese-Cambodian deep-water port project was developed, Koh Kong’s economy has been growing and this might be the cause of improved sanitation resulting in reduced prevalence of *H. pylori* infection. The other study conducted in Phnom Penh, Cambodia’s capital city demonstrated slightly lower *H. pylori* prevalence (26.7%) but almost similar baseline characteristics to our study including female predominance (58.2%) and mean age of 43.9 years in the positive culture group (Tuan et al., 2019). Tuan et al. study revealed higher antibiotic resistance rates of metronidazole (96.4%), levofloxacin (67.3%), and multidrug (76.4%) but lower rate of clarithromycin (25.5%) than our study. However, we did not observe any amoxicillin resistance, whereas there were 9.1% of amoxicillin resistance in the previous study. Multidrug-resistant strains in Tuan et al., (2019) study demonstrated the highest prevalence of resistance to MNZ and quinolone (40.0%), followed by triple resistance to MNZ, CLR, and quinolone (18.2%) which were comparable to our study resistance rates of 35.7%, and 14.3%, respectively. In conclusion, *H. pylori* strains in the capital city exhibited higher antibiotic resistance than strains from rural area. Higher resistance rates in urban area compared to rural areas were also observed in previous studies in Thailand (Vilaichone et al., 2013; Vilaichone et al., 2018).

Compared to other countries in Southeast Asia, this study revealed approximately the same *H. pylori* prevalence as in eastern Thailand, which was the Thai region sharing main boundaries with Cambodia (Vilaichone et al., 2018). Our study disclosed the extremely high prevalence of fluoroquinolone-resistant *H. pylori* strains and also considerably higher than other countries in the same region (Savoldi et al., 2018). Moreover, MNZ and CLR resistance were among the highest in Southeast Asia (Vilaichone et al., 2018). Abundant quinolone resistance in Cambodia was probably because of antibiotic use in treatment for common infectious diseases. Lower respiratory infection was the second leading cause of deaths while diarrhea was also the leading cause of mortality in children under 5 years old in Cambodia (Centers for disease control and prevention, 2018; Merali et al., 2018). Diarrhea and pneumonia were commonly treated by quinolones and this could consequently induce high quinolone resistance rate. In addition, over-the-counter antibiotic dispensing in pharmacies could contribute to increased antibiotic resistance rates in Cambodia. At present, *H. pylori* antibiotic resistance is a substantial global issue resulting in treatment failure in many countries (Savoldi et al., 2018). In 2018, CLR-resistant *H. pylori* ranked first for community-acquired organisms on the WHO priority list of antibiotic-resistant bacteria in search of new antibiotic development to combat them (Tacconelli et al., 2018). Standard triple therapy containing CLR is not recommended to be used if CLR resistance is greater than 15% (Malfertheiner et al., 2017). CLR resistance of 27.8% in our study suggested that standard triple therapy should not be used as first-line treatment for *H. pylori* infection in this country. In contrast, even though there was MNZ resistance, successful eradication could be achieved by extending duration of therapy and addition of other effective antibiotics (Vilaichone et al., 2015). 

CYP2C19 is an important host factor regulating metabolism of all proton pump inhibitors (PPI) causing variability in *H. pylori* treatment outcomes. According to US Food and Drug Administration (USFDA), serious drug interactions can occur attributed to concomitant use of a drug interfering CYP2C19 metabolism such as PPI and a drug requiring metabolism into its active form such as clopidogrel especially in CYP2C19 poor metabolizers. However, there was no prior study of CYP2C19 genotyping in Cambodia. CYP2C19 genotyping in our study revealed majority of rapid and intermediate metabolizers which were comparable to the number of metabolizers in the previous Thai study (Prasertpetmanee et al., 2013). Larger clinical study with proper *H. pylori* treatment regimen should be performed in the future to clarify important role of CYP2C19 genotype on *H. pylori* eradication.

*H. pylori* infection remain common infection in Cambodia. Clarithromycin, levofloxacin, metronidazole and multidrug resistance of *H. pylori* strains are major problems in Koh Kong, Cambodia. The treatment regimen without clarithromycin and quinolone such as 14-day bismuth-based quadruple therapy might be an appropriate choice for *H. pylori* eradication in this particular area and might be helpful to decrease fatal *H. pylori*-associated diseases especially gastric cancer in this country.
